# *Helicobacter pylori* Genotyping from American Indigenous Groups Shows Novel Amerindian *vacA* and *cagA* Alleles and Asian, African and European Admixture

**DOI:** 10.1371/journal.pone.0027212

**Published:** 2011-11-03

**Authors:** Margarita Camorlinga-Ponce, Guillermo Perez-Perez, Gerardo Gonzalez-Valencia, Irma Mendoza, Rosenda Peñaloza-Espinosa, Irma Ramos, Dangeruta Kersulyte, Adriana Reyes-Leon, Carolina Romo, Julio Granados, Leopoldo Muñoz, Douglas E. Berg, Javier Torres

**Affiliations:** 1 Unidad de Investigacion en Enfermedades Infecciosas, Instituto Mexicano del Seguro Social, Mexico D.F., Mexico; 2 School of Medicine, New York University, New York, New York, United States of America; 3 Unidad de Investigacion en Genetica Humana, Instituto Mexicano del Seguro Social, Mexico D.F., Mexico; 4 Department of Molecular Microbiology, Washington University School of Medicine, St. Louis, Missouri, United States of America; 5 Instituto Nacional de Pediatria, Secretaria de Salud, Mexico D.F., Mexico; 6 Instituto Nacional de la Nutricion y Ciencias Medicas, Secretaria de Salud, Mexico D.F., Mexico; Universidad Nacional, Heredia, Costa Rica

## Abstract

It is valuable to extend genotyping studies of *Helicobacter pylori* to strains from indigenous communities across the world to better define adaption, evolution, and associated diseases. We aimed to genetically characterize both human individuals and their infecting *H. pylori* from indigenous communities of Mexico, and to compare them with those from other human groups. We studied individuals from three indigenous groups, Tarahumaras from the North, Huichols from the West and Nahuas from the center of Mexico. Volunteers were sampled at their community site, DNA was isolated from white blood cells and mtDNA, Y-chromosome, and STR alleles were studied. *H. pylori* was cultured from gastric juice, and DNA extracted for genotyping of virulence and housekeeping genes. We found Amerindian mtDNA haplogroups (A, B, C, and D), Y-chromosome DYS19T, and Amerindian STRs alleles frequent in the three groups, confirming Amerindian ancestry in these Mexican groups. Concerning *H.pylori cagA* phylogenetic analyses, although most isolates were of the Western type, a new Amerindian cluster neither Western nor Asian, was formed by some indigenous Mexican, Colombian, Peruvian and Venezuelan isolates. Similarly, *vacA* phylogenetic analyses showed the existence of a novel Amerindian type in isolates from Alaska, Mexico and Colombia. With *hspA* strains from Mexico and other American groups clustered within the three major groups, Asian, African or European. Genotyping of housekeeping genes confirmed that Mexican strains formed a novel Asian-related Amerindian group together with strains from remote Amazon Aborigines. This study shows that Mexican indigenous people with Amerindian markers are colonized with *H. pylori* showing admixture of Asian, European and African strains in genes known to interact with the gastric mucosa. We present evidence of novel Amerindian cagA and vacA alleles in indigenous groups of North and South America.

## Introduction

*H. pylori* infects about 50% of the human population, colonizing the gastric epithelial mucosa for decades and in most cases causing an asymptomatic mucosal inflammatory response. However, a fraction of infected people develop peptic ulcer, gastric carcinoma or MALT-lymphoma. Epidemiological studies suggest that *H. pylori* infection is acquired early in life and although transmission occurs preferentially within families, some studies suggest transmission from other households and even environmental sources in developing country settings. This very mode of transmission suggests that *H. pylori* may have evolved mechanisms to adapt to its different hosts in various human ethnic groups. In fact, considerably evidence shows that *H. pylori* has co-evolved with humans for thousand of years and has been intimately associated with modern humans, even before migration out of Africa some 50 to 70,000 years ago [1]. *H. pylori* displays a genetic diversity that reflects geographic and ethnic separation between the human groups it colonizes. Analyses of genetic diversity and genotyping of *H. pylori* isolates is often accomplished by comparing sequences of several housekeeping or virulence associated genes, or by analyzing total gene content with whole genome-microarrays. In particular studies of sequences of seven housekeeping genes in isolates from different regions of the world showed that *H. pylori* can be divided in seven populations and subpopulations [2]. In addition, different predominant allele types of *H. pylori* virulence genes such as *vacA, cagA, hspA* and *oipA* are associated with different human ethnic groups [3], [4], [5]. For instance, *vacA* signal sequence *s1c* type is found mainly in *H. pylori* from East Asia but not Africa, Europe, or the Americas, whereas *s1b* is commonly found in *H. pylori* from Spain, Portugal and Latin-America. Likewise, the 3’ region of the *cagA* gene is highly polymorphic, and this translates in distinct patterns at the carboxy terminal of the protein. The term “ABD” has been used to define the amino acid sequences flanking EPIYA (tyrosine phosphorylation) motifs that are prevalent in East-Asian isolates; whereas an ABC pattern is typical of most *H. pylori* from Western regions. In addition, an inserted segment, ins180, is more frequent in African and African-American strains than in others [6], especially East Asian strains. Studies on the population genetics of *H. pylori* had been helpful in unraveling human migrations such as those which peopled the Americas [3]. Although some authors have suggested that sensitivity of *H. pylori* genotyping might be even superior to human markers to distinguish geographically related populations [7], recent advances in tests to distinguish human populations, have improved considerable human typing.

Previous reports studying *H. pylori* phylogeny generally have not characterized their human host [2], [3], which is particularly relevant when studying indigenous groups, whose exact ancestries may not be known. There is also a need to extend these studies to more diverse indigenous communities across the world to better define adaption, evolution, and disease associated with this infection. Thus, the aim of our study was to genetically characterize both human individuals and their *H. pylori* isolates from different indigenous groups of Mexico and to compare their virulence and housekeeping genes with those of *H. pylori* strains from other human populations.

## Results

A total of 208 volunteers who still spoke their native language (Table 1) were screened for this study. Of these only individuals with an O-Rh+ blood group phenotype were included to reduce the probability of including outmixed (mestizo) individuals. In the end 191 individuals (91.8%), each from a different family were selected for analyses. One individual sampled in the Huichol community turned out to be native from the Otomi group and was included in the study (23O).

**Table 1:** Native language and frequency of mtDNA haplogroups in the Mexican Indigenous populations of this study, contrasted with previous studies in Mexican and other populations.

		Language		mtDNA Haplogroups, percent				
Population	No. studied	Native language	Linguistic Family	A	B	C	D	other
*This study:*								
Nahua	105	Náhua	Uto-azteca	**37.1**	29.5	26.7	6.7	0
Huichol	14	Huichol	Uto-azteca	14.3	**50.0**	35.7	-	-
Tarahumara	68	Tarahumara	Uto-azteca	13.2	**39.7**	**36.7**	8.8	1.5
*Previous studies:*								
Nahua Atocpan^a^	59	Náhua	Uto-azteca	13.6	**35.6**	11.8	2.0	0
Otomi SnAnt^b^	38	Otomi	Otomanguean	**39.5**	13.2	**39.5**	2.6	5.2
Mexican Mestizo^c^	270	Spanish	Spanish	**51.1**	17.8	18.5	5.9	6.7
*Other populations* d								
Caucasus				1	0.5	4	4	**90.5**
East Asia				7	16	5	**26**	**46**
Africa				0	0	0	0	**100**

a Peñaloza-Espinosa et al. [11];

b Sánchez-Boiso et al. [12];

c Guardado-Estrada et al. [13];

d http://www.mitomap.org/bin/view.pl/MITOMAP/HaplogroupMarkers [14].

### Amerindian mtDNA, Y-chromosome, and STRs genotypes are highly frequent in the three Native groups

Typical Amerindian mtDNA haplogroups (A, B, C, and D) were found in the Mexican Natives studied, with some differences among groups. Haplogroup A was at higher (35%) frequency in Nahuas, than in Huichols and Tarahumaras, whereas haplogroups B and C were more common among Huichols and Tarahumaras. Haplogroup D has a generally low prevalence (Table 1). In addition, we found one individual from the Tarahumara group with haplogroup X, which is common in some natives from North America, especially Alaska [8]–[9]. Table 1 also describes frequencies of mtDNA haplogroups in Mexican and other groups reported previously and the previous data are in accord with ours, and emphasizes that Mestizo, Caucasian and Asian groups tend to differ in these mtDNA markers [10], [11], [12], [13].

Among Y-chromosome markers a higher frequency of DYS19T (characteristic of Amerindians) was observed in the Huichol and Nahua than in the Tarahumara group (Table 2). Previous studies have also reported higher frequency of this allele in Huichol and Nahua than in Tarahumara groups and also shown it to be even lower in Mestizo groups (Table 2) [14], [15]. The DYS19T allele frequency is very low in Europe, and moderately low in North Asia (Table 2) [16]. Thus, our results document Amerindian markers in all three populations, with the Huichol showing evidence of higher genetic isolation than the Tarahumara and Nahua groups.

**Table 2:** Frequency of polymorphisms in DYS19T of the Y-chromosome in Native and Mestizo Mexican Populations and other populations.

Population	Num. studied	DYS19T (%)
*This study:*		
Nahua	31	74.2
Huichol	4	100
Tarahumara	21	33.3
*Previous studies:*		
Huichol a	34	100
Tarahumara^a^	20	55.0
Nahua^a^	34	79.4
Guerrero/Mestizos^b^	4	25.0
Western/Mestizo^a^	191	17.3
*Other populations* c		
Native Americans	588	76.4
Europe	237	0.4
North Asia	669	17.9

a Rangel-Villalobos et al. [15];

b Bonilla C. et al [16];

c Zegura et al [17].

To further characterize these groups, we also studied 15 informative STRs alleles. The alleles most frequent in our three Mexican groups also are typical Amerindian populations (Table 3). The frequencies of the STR alleles found in this study are in agreement with previous studies in Mexican Native communities [17], [18], this is further detailed in Table S1. Of special interest to us were STR markers of persons colonized with Asian-related *H. pylori* strains marked in grey in Table 3, one Huichol (368H), one Otomi (23O) and one Tarahumara (590T). Table S1 shows that their STR alleles were not among the most frequent in these groups, and confirms that these three individuals have Amerindian markers uncommon in the rest of the aborigines studied.

**Table 3:** Frequencies of STRs alleles in three Mexican Native populations*.

Allele	D8S1179	D21S11	D7S820	CSF1PO	D3S1358	D13S317	D16S539	D2S1338	D19S433	D18S51	D5S818	FGA
6.3	--	--	--	--	--	--	--	--	--	--	--	--
7	--	--	--	--	--	--	--	--	--	--	0.1333	--
8	--	--	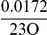	--	--	0.0333	--	--	--	--	--	--
9	--	--	--	--	--	0.3000	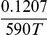	--	--	--	0.0167	--
9.3	--	--	--	--	--	--	--	--	--	--	--	--
10	0.1587	--	0.1552	0.1964	--	0.2167	0.2241	--	--	--	0.0333	--
11	0.0317	--	0.4310	0.1607	--	0.2000	0.1379	--	--	0.0172	0.5667	--
12	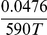	--	0.3276	0.5714	--	0.0833	0.3793	--	--	0.0345	0.1500	--
12.2	--	--	--	--	--	--	--	--	--			--
13	0.3810	--	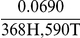	0.0357	--	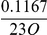	0.1379	--	0.1667	0.2069	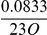	--
13.2	--	--	--	--	--	--	--	--	0.2000	--	--	--
14	0.2540	--	--	0.0357	0.0167	0.0500	--	--	0.1500	0.2069	--	--
14.2	--	--	--	--	--	--	--	--	0.0500	--	--	--
15	0.1111	--	--	--	0.5500	--	--	--	0.1500	0.1552	--	--
15.2	--	--	--	--	--	--	--	--	0.1000	--	--	--
16	--	--	--	--	0.3333	--	--	--	--	0.0690	--	--
16.2	--	--	--	--	--	--	--	--	0.1167	--	--	--
17	0.0159	--	--	--	0.1000	--	--	0.0500	--	0.2241	--	--
17.2	--	--	--	--	--	--	--	--	0.0667	--	--	--
18	--	--	--	--	--	--	--	0.1000	--	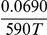	--	--
19	--	--	--	--	--	--	--	0.1667	--	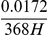	--	0.2414
20	--	--	--	--	--	--	--	0.1833	--	--	--	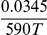
21	--	--	--	--	--	--	--	0.0167	--	--	--	0.0862
22	--	--	--	--	--	--	--	0.1667	--	--	--	0.1207
23	--	--	--	--	--	--	--	0.2167	--	--	--	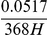
23.2	--	--	--	--	--	--	--	--	--	--	--	--
24	--	--	--	--	--	--	--	0.0833	--	--	--	0.1897
25	--	--	--	--	--	--	--	0.0167	--	--	--	0.0862
26	--	--	--	--	--	--	--	--	--	--	--	0.1379
27	--	0.0161	--	--	--	--	--	--	--	--	--	0.0345
28	--	0.0161	--	--	--	--	--	--	--	--	--	0.0172
29	--	0.2097	--	--	--	--	--	--	--	--	--	--
30	--	0.2742	--	--	--	--	--	--	--	--	--	--
30.2	--	0.0161	--	--	--	--	--	--	--	--	--	--
31	--	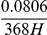	--	--	--	--	--	--	--	--	--	--
31.2	--	0.1774	--	--	--	--	--	--	--	--	--	--
32.2	--	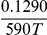	--	--	--	--	--	--	--	--	--	--

*Letters in bold mark the more frequent alleles; in italics are the alleles distinguishing the three Native strains, 368H, 23O, and 590T.

### vacA and cagA alleles of H. pylori from native Mexican communities

In total we recovered and genetically analyzed *H. pylori* from 35 study participants: 18 Nahuas, 11 Tarahumaras, 5 Huichols and one Otomi (living in a Huichol community) (Table 4), and the GenBank accession numbers of *vacA* and *cagA* sequence are in Table S2. At the vacA locus, most strains contained European-type s1b signal sequence and m1 mid region alleles. However, four of the five Huichol isolates were s1b m2, and a few Nahua and Tarahumara isolates (4/18, 3/11, respectively) were of the putatively less virulent vacA s2 m2 type (Table 4). We also note, that one exceptional Huichol isolate (368H) contained an s1c-type allele.

**Table 4:** Distribution of *vacA* alleles in *H. pylori* strains isolated from Mexican indigenous populations.

vacA alleles, No. of strains						
Native population	s1am1	s1bm1	s1bm2	s1cm2	s2m2	Total
Nahua	2	13*	-	-	4	18
Huichol	-	-	4	1	-	5
Tarahumara	-	7	1	-	3	11
Otomi	-	-	1	-	-	1
Total (%)	2 (5)	20 (57)	6 (17)	1 (3)	7 (20)	36

*One of these strains (172N) presented a slabm1 allele.

**Figure 1:**
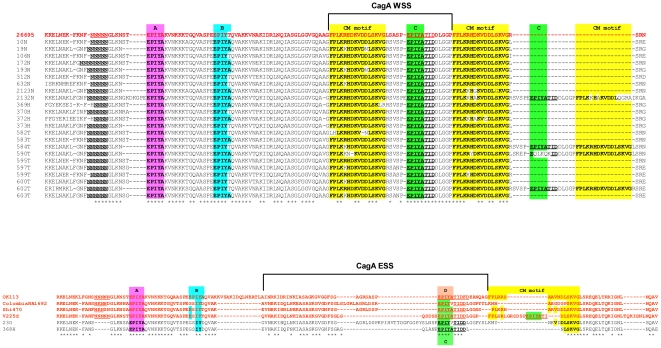
Alignment of amino acid sequences of the CagA 3’ region of *H.pylori* strains from Mexican native groups. a) Most strains presented a Western-type sequence, with an EPIYA ABC pattern and two CM motifs. The sequence of strain 26695 was used as reference. b) One Huichol strain, 368H and one Otomi strain, 23O presented a chimeric sequence with some homology with Asian-type sequences, with insertions, deletions and substitutions and similar to the Colombian strain NA1692. Sequences are compared with the Japanese isolate OK113.

All but four of our 35 isolates contained a *cagA* virulence gene, which, in most cases seemed to be Western-like in sequence at the critical carboxy terminal region, although it was generally smaller than that reported by others in Mexican-Mestizo isolates [19]: 492 to 615 codons vs. 500 to 850 codons in ref 20. In fact, most of the CagA proteins encoded in our strains contained just three EPIYA-type repeats and one CM motif (Table 5). Several strains (ten) contained variant EPIYT B-type (Figure 1) and/or variant CM motifs (most often, K, not R, at position 5, Figure 1). A few strains, especially from Tarahumara people, contained two CM motifs (Figure 1). Of particular note were two isolates with a variant GSIYD B motif, a distinctive D-like motif and a partially deleted CM motif -- strains 23O and also 368H, which also contains *vacA s1c*-type allele (Figure 1). This type of peculiar CagA sequence has also been reported in strain NA1692 from an indigenous Colombian (Figure 1).

**Table 5:** Characteristics of 35 individuals from Mexican indigenous groups and their *H. pylori* isolated strains.

No. individual	Origin	age	sex	*vacA*	cagA	EPIYA pattern	hspA
632N	Nahua	32	m	s1bm1	+	ABCC	A2
10N	Nahua	68	f	s1bm1	+	ABC	A2
11N	Nahua	75	f	s2m2	-	-	A1
111N	Nahua	78	m	s2m2	-	-	A1
172N	Nahua	49	f	s1bm1	+	ABC	A1
1831N	Nahua	29	f	s1bm1	+	ABC	A1
19N	Nahua	81	f	s1bm1	+	ABC	A1
193N	Nahua	33	m	s1bm1	+	ABC	A1
50N	Nahua	43	m	s1bm1	+	ABC	A1
203N	Nahua	20	m	s1bm1	+	ABC	A1
2123N	Nahua	42	f	s1bm1	+	ABC	A2
2132N	Nahua	21	m	s1bm1	+	ABCC	A1
2133N	Nahua	17	f	s1bm1	+	ABC	A1
58N	Nahua	16	m	s2m2	-	-	A2
2161N	Nahua	27	m	s2m2	-	-	A2
2921N	Nahua	25	m	s1am1	+	ABC	A2
312N	Nahua	20	f	s1bm1	+	ABC	A2
35N	Nahua	48	f	s1bm1	+	ABC	A1
580T	Tarahumara	80	m	s1bm1	+	ABC	A2
582T	Tarahumara	52	f	s1bm1	+	ABC	A1
584T	Tarahumara	48	m	s1bm1	+	ABCC	A2
590T	Tarahumara	26	m	s2m2	+	ABC	A1
594T	Tarahumara	32	f	s1bm1	+	ABC	A1
595T	Tarahumara	16	f	s1bm1	+	ABC	A1
597T	Tarahumara	28	f	s1bm1	+	ABC	A1
599T	Tarahumara	18	m	s1bm2	+	ABC	A1
600T	Tarahumara	80	f	s1bm1	+	ABC	A1
602T	Tarahumara	49	f	s2m2	+	ABCC	A2
603T	Tarahumara	78	f	s2m2	+	ABCC	A1
373H	Huichol	32	f	s1bm2	+	ABC	A2
370H	Huichol	20	f	s1bm2	+	ABC	A2
369H	Huichol	40	m	s1bm2	+	ABC	A2
372H	Huichol	43	m	s1bm2	+	ABC	A1
36H8	Huichol	47	m	**s1cm2**	+	**ABD**	A2
23O	Otomi	25	F	s1bm2	+	**ABD**	A1

### Phylogenetic analyses suggest the existence of specific cagA and vacA Amerindian clusters

**Figure 2:**
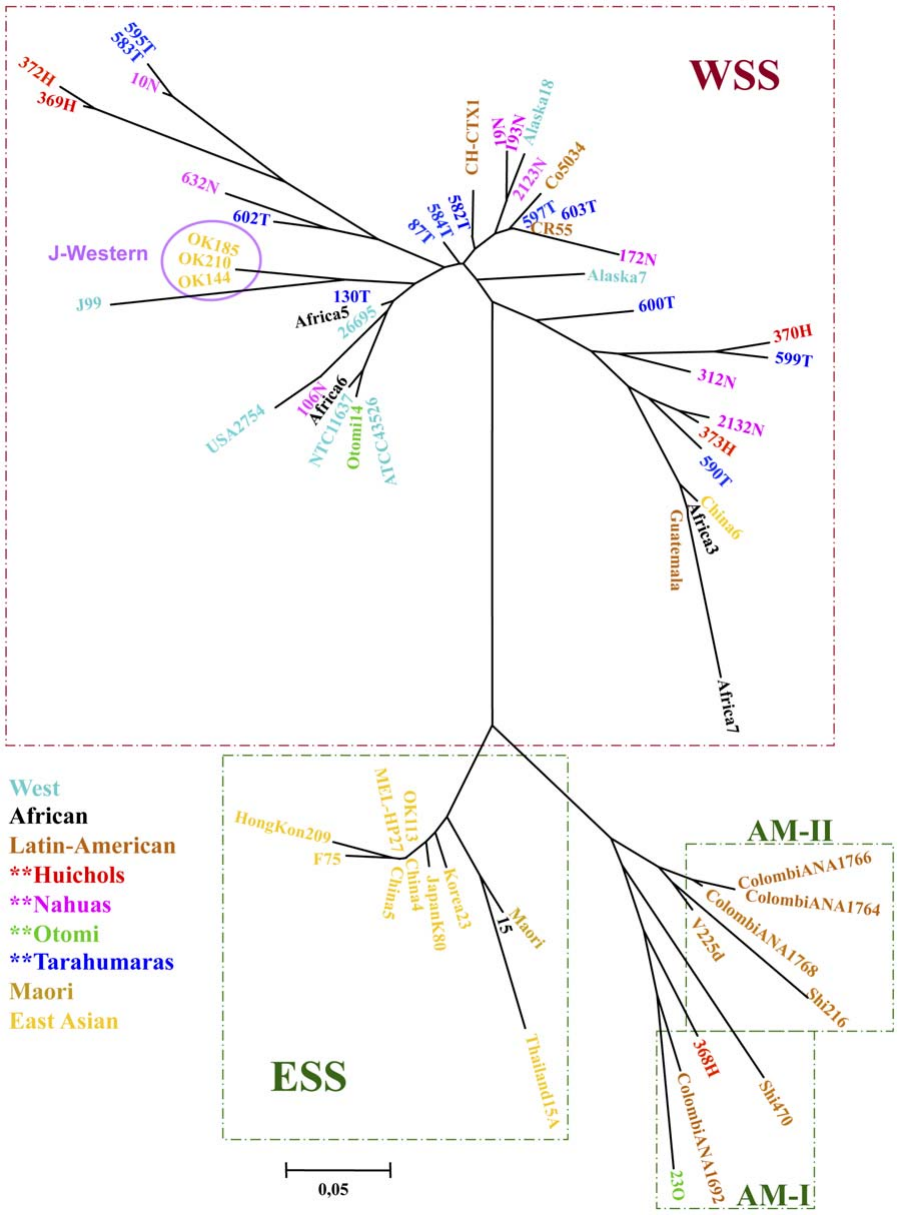
Phylogenetic analysis of sequences of *vacA* of *H. pylori* strains from Mexican native groups. The trees were constructed by the Neighbor-joining method based on Kimura's two-parameter model distance matrices. Phylogenetic analysis of *vacA* signal sequence shows most sequences clustered in the s1b group, whereas the Otomi 23O strain together with some Colombian isolates grouped between s1a and s1b. The Huichol 368H grouped close to the s1C branch, together with some Alaskan and Colombian strains.

Phylogenetic analyses of *vacA* confirmed the grouping of 368H Huichol strain outside the West s1a and s1b clusters, and placed it in a cluster related to, but not within the East-Asian group (Figure 2). Interestingly, this cluster included also isolates from indigenous communities of Alaska, Colombia and Peru, indicating the existence of a novel *vacA* Amerindian cluster. In contrast, strain 23O, which also seemed Asian like in its *cagA* D motif, contained a Western-like *vacA* s region allele, as did several Colombian isolates (NA1764, NA1766 and NA1768 in Figure 2).

**Figure 3:**
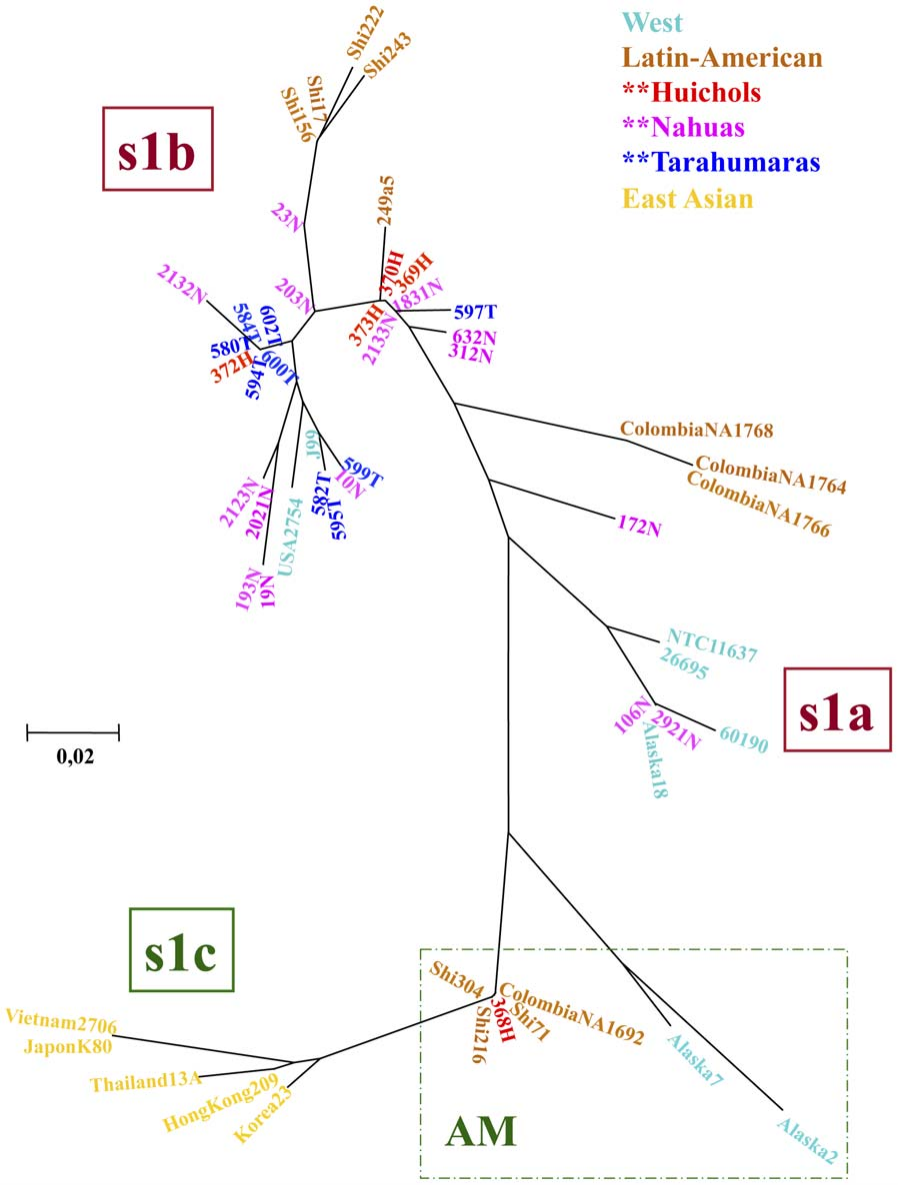
Phylogenetic analysis of sequences of *cagA* of *H. pylori* strains from Mexican native groups. The trees were constructed by the Neighbor-joining method based on Kimura's two-parameter model distance matrices. Phylogenetic analyses of the 3′ region of *cagA* shows that whereas most isolates clustered in the Western group (WSS), the Huichol 368H and the Otomi 23O strains grouped closer to the East-Asian group (ESS), together with some Colombian strains.

In the phylogenetic analyses of *cagA*, most Mexican indigenous isolates were in the Western group, except for the Huichol 368H and Otomi 23O strains, which clustered in a group related to the East-Asian group (Figure 3). This was expected since the *cagA* sequence of these strains presented a mixture of Western and East-Asian motifs. It is noteworthy that indigenous isolates from Colombia, Peru and Venezuela, clustered with this two unusual isolates forming two subgroups (Figure 3), like those recently described and designated as AM-I and AM-II, for Amerindian subgroups [20].

### Phylogenetic analyses of hspA show evidence of Asian or African ancestry for some Mexican isolates

**Figure 4:**
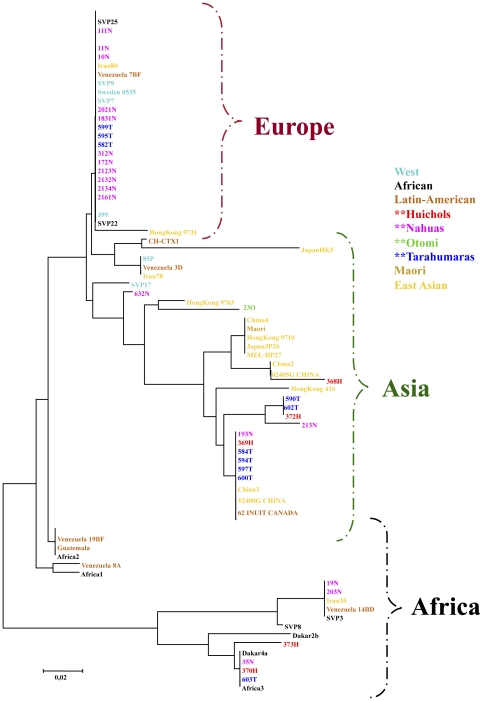
Phylogenetic analysis of sequences of *hspA* of *H. pylori* strains from Mexican native groups. The trees were constructed by the Neighbor-joining method based on Kimura's two-parameter model distance matrices. Phylogenetic analyses of *hspA* shows that whereas many of the Nahua strains clustered in the Europe group, many of the Tarahumara isolates clutered in the Asia group together with Huichol strains. Some isolates also grouped in the Africa group.

Phylogenetic analysis of *hspA* showed that isolates from the three Mexican indigenous groups were present in all, Asian, European and African groups (Figure 4). Still, Nahuas were more prevalent in the Europe group and Tarahumara in the Asian group. In addition, there were strains from native groups of Canada and Venezuela, which also grouped within the Asian cluster. A few isolates from the three Mexican communities clustered within the African group, together with isolates from aboriginals of Venezuela, Guatemala and Brazil.

Consistent with the phylogeography observed with *cagA* and *vacA*, the indigenous strains 368H and 23O clustered within the Asia group of hspA (Figure 4).

### Results with the MLST analyses confirmed the existence of an Asian-related Amerindian H. pylori group

**Figure 5:**
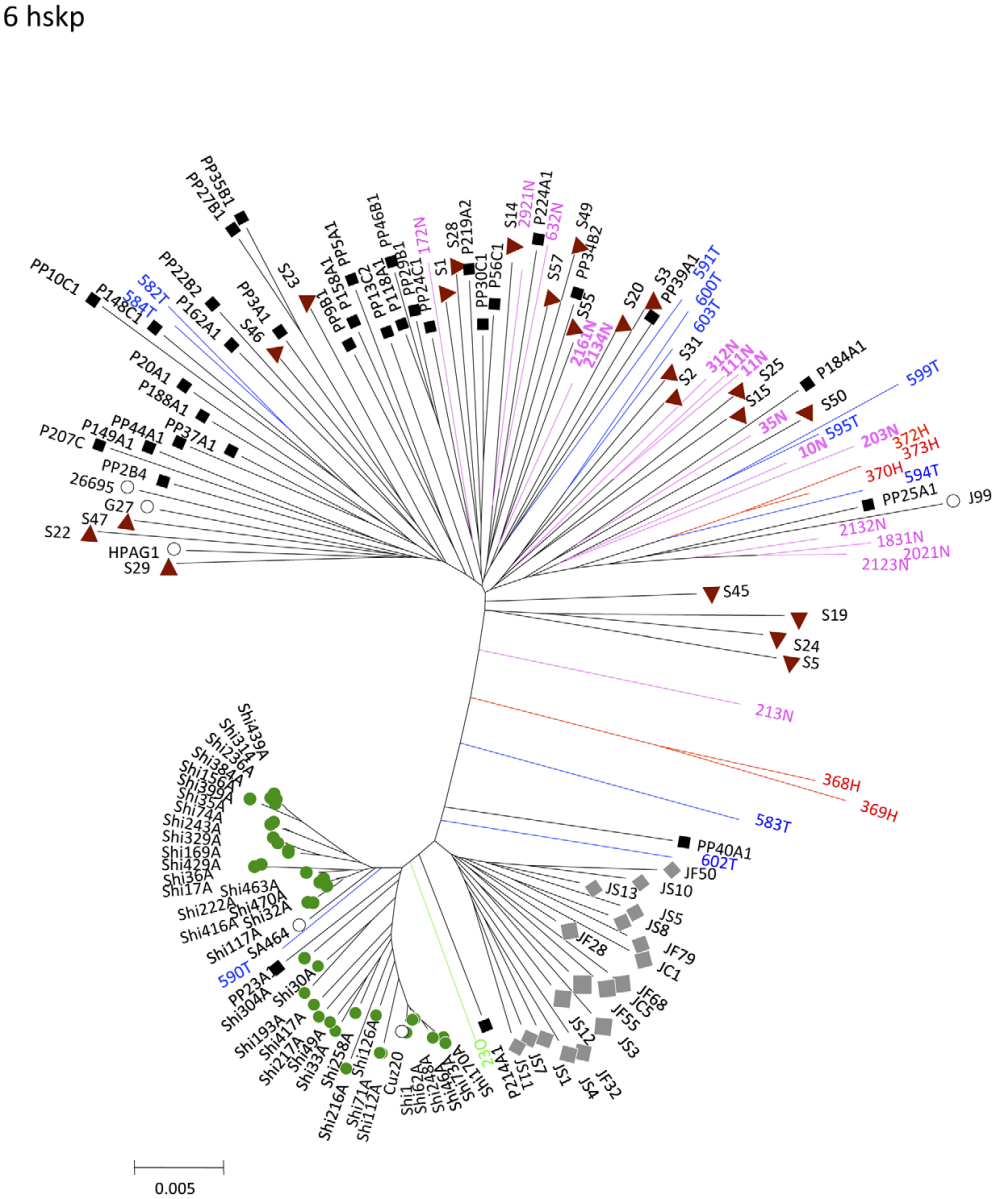
Phylogenetic analyses of concatenated six housekeeping genes (Multilocus sequence typng). Results show that although many of the Mexican isolates clustered within the European group, two strains (23O and 590T) clustered within a group closely related to the East Asian, together with other *H. pylori* isolates from Indigenous Amazon people from Peru which is now identified as a novel Amerindian group.

As depicted in Figure 5, the MLST analyses of concatenated six housekeeping genes showed that many of the Mexican isolates clustered within the European group, as did isolates of mestizo population from other Latin-American countries. However, the analyses also showed that two strains, one from the Otomi (23O), and one from the Tarahumara (590T) natives, clustered within a group formed by isolates from indigenous Amazons people from Peru, in a place related to but not intermingled with Asian isolates, indicating the existence of an Amerindian group, as recently suggested [21]. These two Mexican native strains clustered in the East-Asia group of hspA (Figure 2c), and 23O present the *cagA* Amerindian type (Figure 1). In the individual analyses of the six housekeeping genes 590T presented the Amerindian allele in the six genes whereas 23O presented Amerindian allele in three genes an Asian in one.

Five other strains, two Huichols, two Tarahumaras, and one Nahual located in a position between the European and the Asian groups (Figure 5), at a unique intermediate genetic distance not observed in any strain from all the other population groups. Of these, the 368H had the *cagA* Amerindian type and an Amerindian allele in two housekeeping genes, whereas strains 602T and 583T presented Asian allele in four and three housekeeping genes respectively.

### Microarray analyses of the Huichol strain with East-Asian ancestry

**Figure 6:**
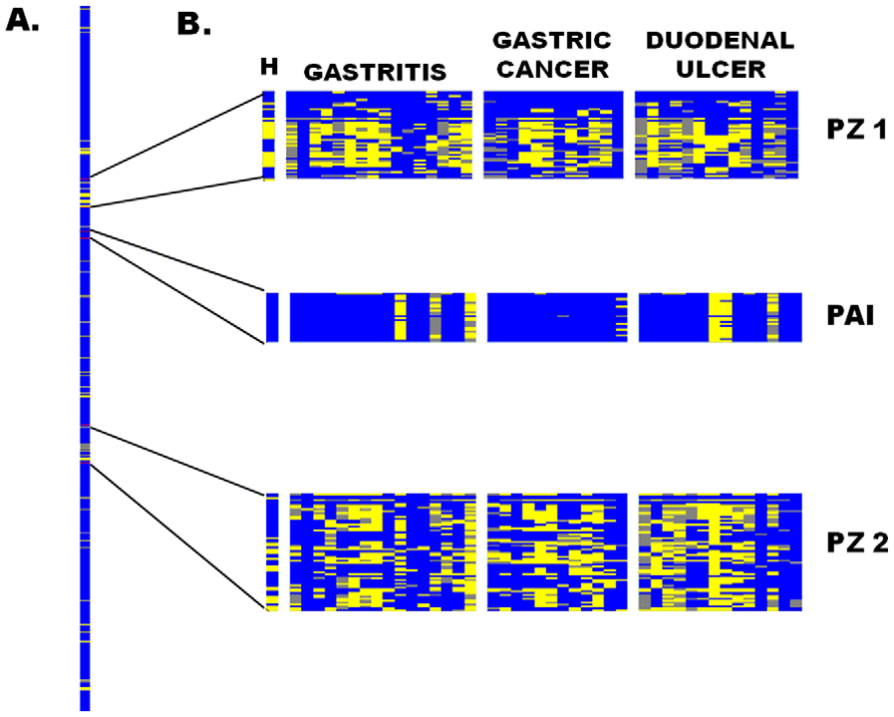
Genomic comparison of the Huichol *H. pylori* 368H strain with strains from Mexican-Mestizo population using whole-genome microarrays. A). Representation of the entire *H. pylori* chromosome of the 368H strain, ordered combining the maps of strains 26695 and J99. The scanogram indicates genes present (blue) or absent (yellow) and missing data (gray). B). The plasticity zones PZ1, PZ2 and cagPAI are compared with that of patients with gastritis, gastrtic cancer and duodenal ulcer. Variability in the gene content of these zones was significantly lower in the Huichol strain.

Because of the peculiar sequences in virulence genes, we explored the genomic content of the *H. pylori* Huichol 368H isolate using microarray based comparative genomic hybridization (a-CGH). Of the 1,660 genes analyzed, 1,539 were present in the isolate (genome core) and 117 (7.04%) genes were absent and were considered as variable genes. Of these 117 variable genes, 41 (35%) were distributed within the previously described plasticity zones (PZs) [22], 21 within the PZ1 and 20 within the PZ2; the remaining 76 genes were scattered along the genome (Figure 6A). Results with the 368H strain were compared with those previously reported in Mexican mestizo groups (Figure 6B). Important differences were found in the variable genes, and the total number of genes absent in 368H was significantly lower than in the mestizo groups (Table S3) and the difference was mostly observed in the PZs and cag PAI (Figure 6B). In fact, as much as 225 genes reported as variable in mestizo groups were present in the Huichol strain, suggesting that in this strain PZs are more conserved, and a concordance analyses showed that PZ1 was the zone with higher disagreement between the mestizo strains and 368H.

### Activity of indigenous strains on AGS cells

**Figure 7:**
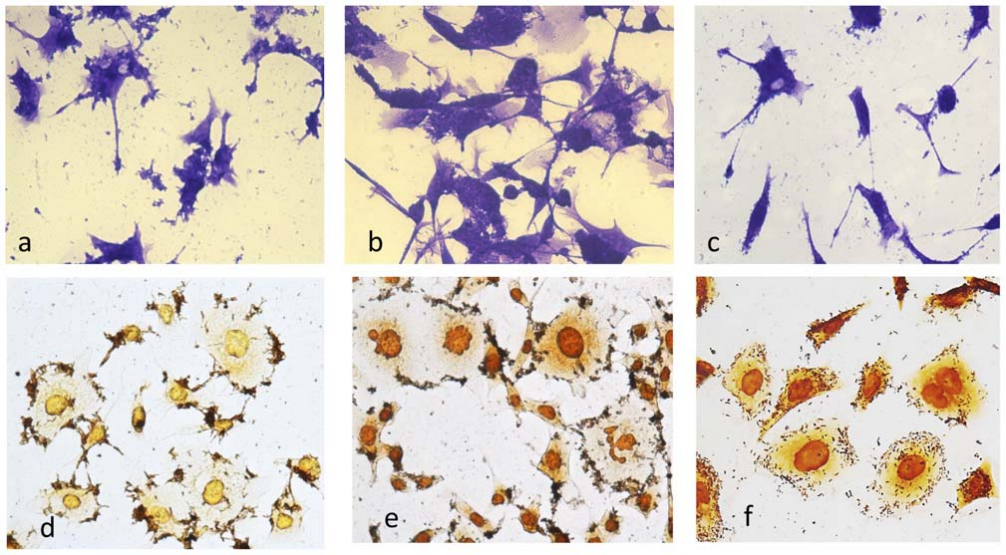
Activity of *H. pylori* strains from Mexican native groups on epithelial gastric AGS cells. We tested the activity of the Huichol strain 368H and of the Otomi 23O on AGS cells after 24 hs of co-culture, and stained with Giemsa: a) AGS incubated with 368H, b) AGS incubated with 23O, c) AGA incubated with a CagA (+) strain from a patient with DU. The pattern of adherence was monitored after 3 hs of co-culture, and stained with Whartin Starry: d) AGS incubated with 368H, e) AGS incubated with 23O, f) AGS incubated with the cagA(+) DU patient. All images were taken at a 40X magnification.

Because of the particular sequence of *cagA* in the 368H and the 23O strain, as well as the difference in gene content observed in microarrays with the 368H strain, we tested the activity of these isolates on AGS cells (Figure 7). Since both strains have a highly modified EPIYA–B motif (GSIYD) and a single CM motif with deletions and partial homology to the Asian motif (Figure 1), we expected a reduced activity on cells. Indeed, whereas 368H and 23O caused a poor elongation after 24 hs of incubation (Figure 7a and 7b), the strain from a Mexican mestizo caused a marked cell elongation (Figure 7c). The two strains were able to adhere to the cell surface after 6 hs of co-culture, although the pattern of adherence of both strains differed from that observed in isolates from a Mestizo individuals; 368H and 23O strains formed micro-colonies around the cell (Figure 7d and 7e), whereas bacteria of the other Mestizo strain scattered across the AGS surface (Figure 7f). Both 368H and 23O strains caused a marked induction of IL-8 (666 and 681 pg/ml, respectively). We tested two Tarahumara strains that were cagPAI negative (594T and 582T), both adhered to the cell with a scattered pattern, but none were positive for hummingbird effect or IL-8 induction (results not shown).

## Discussion

### Evidences of mtDNA and Y-chromosome confirm a mixture of Asian-Amerindian and African ancestry in the native groups studied

In this work we aimed to learn about ancestry in the native groups studied for *H. pylori* infection in order to genetically characterize both, the host and the bacteria. It is known that the Native American groups are usually characterized by different proportions of mtDNA haplogroups A, B, C, D and X [23], [24]. In the present work, we found the presence of all mtDNA Amerindian haplogroups reported in both, North and South America [9], which confirms a Native-American maternal ancestry in the three Mexican groups studied. We found the haplogroup B as the most frequent, followed by haplogroup C, haplogroup A and haplogroup D, respectively. We found the haplogroup X only in one Tarahumara individual, an haplogroup which has been reported in some native groups of North America [10]. This finding confirms previous reports on the presence of this uncommon haplogroup in a few individuals of the same Tarahumara groups, which live in the North part of Mexico [25].

For the polymorphisms in the Y-chromosome, the DYS19T marker was the most frequent in the Mexican groups studied, similar to reports in other Native American communities [17]. This polymorphism was present in all Huichol individuals tested (West of Mexico), in most of the Nahua population (Center of Mexico), and to a lesser extent in the Tarahumara group (North of Mexico). These frequencies are similar to previous studies in Mexican native and mestizo groups, and confirm the admixture of pre-Spaniard native Mexican communities with European population, and to a much lesser extent with African markers [26]. The frequency of STRs alleles in the populations studied also demonstrated similarities with previous studies in Mexican Natives [18], [19]. Thus, genotyping of individuals from the three Mexican native groups studied document that although all have Amerindian markers, each population is different based on the frequency of mtDNA, Y-chromosome and STRs markers. Although these are not novel findings, the relevant fact in our study is that we confirmed that the indigenous Mexican groups studied conserved many Amerindian alleles. Still, we should note that no significant differences are observed when contrast with Mexican mestizo population, particularly with the STRs alleles, a finding which has been reported previously [18] and which suggest that Mexican mestizo population still conserve many Amerindian genetic markers. A few strains from the Tarahumara and Huichol groups had Amerindian STRs alleles that were uncommon in the rest of the population studied, which is in agreement with the fact that these strains presented also Amerindian types in housekeeping and/or virulence genes (see below).

### Polymorphisms in virulence genes vacA and cagA point to a discrete Amerindian type

Similar to results with the human genotypes, polymorphisms in virulence genes of the *H. pylori* infecting strains also differed among the populations. Thus, in the Nahua group the most prevalent *vacA* allele was s1bm1, which is similar to what we previously observed in Mexican mestizo groups [27] and it is also the genotype more often observed in populations of Latin America. This observation is also in accordance with the fact that in the Nahua group we observed the higher frequency of the mtDNA haplogroup A, which is also the most prevalent in Mexican-mestizo groups and reflects the outmix of Spaniards and Native Americans [26]. In contrast, the *vacA* haplogroup s1m2 was highly prevalent in the Huichol group, which also presented the higher frequency of the mtDNA haplogroup B. In addition, the 368H Huichol strain presented an s1c-related allele, characteristic of East-Asian *H. pylori*. These results were confirmed with the phylogenetic analyses of the s region sequence, where we observed that 368H grouped in a cluster closely related to the East-Asian group. Furthermore, in this Asian-like group were included strains from Natives of Alaska, Mexico, Peru and Colombia and represent a novel vacA Amerindian group not previously described.

Concerning CagA, most of the indigenous Mexican isolates shared homology with a Western type sequence, with most strains having an ABC-2CM motifs pattern; which contrasts with the ABCC-3CM pattern which we previously observed in several isolates from Mexican-mestizos living in Mexico City [20]. However, two isolates, one from Huichol (368H) and one from Otomi (23O) groups presented a chimeric CagA 3’ region with partial homology with the East-Asian D motif and with one partially deleted CM motif, which showed segments of partial homology with both, the Western and the East-Asian CM motif. Of note, some *H. pylori* isolates from indigenous Colombia, Venezuela and Peru groups [4], [28] also presented a chimeric 3′region with high homology with the 368H and 23O Mexican isolates, showing evidences of similar Western-Asian recombination in *H. pylori* Amerindian groups of both, North America and South America. In the phylogenetic analyses of CagA, 368H and 23O clustered together with indigenous isolates from Colombia, Venezuela and Peru and formed a group related to, but not intermingled with the East-Asian group, which might represent a novel Amerindian group of CagA, similarly to what we observed in the case of VacA (see above). Independently Suzuki et al. [21] documented specific CagA sequences in strains from the remote Peruvian Amazon that are similar to those reported here, and placed their sequences in two sub-groups designated as AM-I and AM-II. The *cagA* genes of our Mexican Native strains 368H and 23O are of the AM-I sub-group. This supports the view that these unusual alleles were likely widespread in Amerindian populations. Thus, our results confirm the important report by Suzuki et al [21] and extend the finding to isolates from indigenous groups of Mexico and Colombia.

In addition, as indicated above, we also suggest the existence of an Amerindian type of *vacA*, formed by the same strains included in the *cagA* Amerindian group. This would represent a novel group of *cagA* and *vacA* present in strains colonizing indigenous Amerindian groups across America (Amerindian *cagA* and *vacA*). These observations in *H. pylori* strains encompassing North and South America (as exemplified with isolates from Alaska, Mexico, Venezuela, Peru and Colombia) suggest the presence of a discrete group of Amerindian strains with frequent recombination events in *vacA* and *cagA*, most probably the result of adaption to the human groups who populated the Americas some 30,000 years ago [29]. Thus, our results represent current traces of the Asian-Alaska-North America –South America population wave of the Americas.

### Analyses of HspA show admixture of African, Asian and European genes in American Indigenous isolates

*hspA* is informative about African ancestry [5], [6] and is a gene with a vital role as co-chaperone and as a stress response protein [30], [31]. We analyzed this gene and identified traces of African ancestry among a few of the Mexican indigenous strains, and in Native strains from Central and South America, and may represent remnants of the primary African origin of *H. pylori*, documented in previous studies [1], or traces of the migrant African groups which came as slaves. The analyses also showed Asian ancestry for the Amerindian strains 368H, 23O and 590T and for isolates from other Indigenous groups from South America and Canada, which is consistent with our findings in the 3′ region of *cagA*, and provides an independent support for Asian ancestry in Native groups across America. By looking at the phylogenetic pattern of *hspA* it might be suggested that this gene has been more conserved than *vacA* or *cagA* across human migrations, probably because less selective pressure, or because of differences in recombination rates.

### The MLST analyses further support an Asian-related Amerindian group with indigenous strains from Mexico and Peru

The MLST analyses of housekeeping genes have shown to be a robust and consistent test to study ancestry and evolution of *H. pylori* populations [2]. Applied to the Mexican native isolates, although most strains from the three native groups studied were placed within the European group, we also confirmed the Asian ancestry of one Tarahumara and one Otomi strains. Recently, Kersulyte et al. [32] reported that strains from remote Amazon were related to those from Asia, suggesting they descend from *H. pylori* infecting Asian people who migrated to America some 30,000 years ago [29]. Still, isolates from this Shimaa community clustered in a discrete separated group from East-Asia isolates, documenting a separate evolutive adaption to this geographically distant region. Of particular interest is the observation that the two Mexican isolates in the Asian branch also clustered with the discrete group formed by the Shimaa isolates, suggesting a common ancestry for these Amerindian groups across America, and the presence of similar selective forces in these indigenous Latin-American communities located in North America (Mexico) and South America (Peru).

Some Mexican isolates did not clustered with either, the European or the Asian groups, but localized at an intermediate genetic distance, suggesting that they represent Western- Asian recombinant strains, probably in the process to evolve from an Amerindian to a European genetic type. This localization was exclusive for native Mexican strains, not observed in any strain from the other populations studied, and might represent living evidence that Amerindian strains are being displaced by European strains [32].

It should be noticed that the three Mexican strains with Amerindian type in housekeeping genes and virulence *cagA* and *vacA* genes, 590T, 368H and 23O were isolated from indigenous people with Amerindian STRs alleles uncommon in the rest of the Native people studied, which would be in agreement with a selection of strains based on the genetics of the host, leading to the suggested co-evolution of *H. pylori* with human groups.

### Gene content and activity on cells of the Huichol 368H strains differ from Mexican-mestizo strains

We analyzed the gene content of the 368H strains with the same microarray system we used previously to study Mexican mestizo strains [33], with the main finding being that the variable genes present in mestizo strains were significantly less present in the Huichol strain, mostly in the PZs and cag PAI, which would suggest a modified activity of this strain on epithelial cells. Our results did confirmed a reduced elongation activity of the 368H and 23O strain on AGS cells, which would be in agreement with the fact that these strains have a modified EPIYA–B motif [20]. Still, they were able to induce IL-8 with values similar to those observed in strains from mestizo patients with duodenal ulcer or gastric cancer [20]; similar results were recently reported for the Venezuelan strain v225 d, which presents homology with 368H in the 3′region of CagA [28]. Both strains were able to bind and reproduce on epithelial cells, although using an adherence pattern different from that observed in other strains from Native or Mestizo Mexican individuals, suggesting important differences in the way they interact with epithelial cells. Thus, in spite of having a modified EPIYA and CM motifs, these strains still displayed activity on AGS cells.

## Conclusions

This study describes genotyping of virulence and housekeeping genes in *H. pylori* strains from Mexican indigenous groups and shows that Mexican Natives with Amerindian genes are infected with *H. pylori* strains with traces of Asian or African ancestors. The study shows novel alleles in *cagA* and *vacA* virulence and in housekeeping genes, particularly in communities genetically more isolated. In addition these Amerindian types were found in strains of indigenous groups from North to South America. These results call for more studies on *H. pylori* strains from Amerindian groups to better understand their co-evolution in the new world races and eventually learn more about this adaption and its consequences for disease.

## Materials and Methods

### Ethics statement

This work was approved by the National Ethics Committee for Research of the Instituto Mexicano del Seguro Social, Mexico. Volunteers were included in the study after they were informed of the nature of the study and signed a consent form.

### Population studied

One hundred and eighty nine individuals from different Mexican indigenous groups were studied, none of the groups were accessible by public transport: 105 Nahuas from San Pedro Tlacotenco and Santa Ana communities nearby Mexico City, reached by not paved roads, but with access to public transportation; 32 Huichol from Sierra de Guadalajara in the West states of Jalisco and Nayarit, reached only by foot trail and no public transportation; and 52 Tarahumaras from Bahuichivo and Bocoina in the North sate of Chihuahua, located in a large canyon reached by uneasy foot trails (Figure S1). In addition, two Otomi native individuals living in the Huichol community were included. People were informed about the nature of the study and those willing to participate signed an informed consent.

### Biological Samples

All biological samples were taken from volunteers at their respective community sites. Four ml of peripheral blood were drawn in a tube with EDTA and kept on ice during transportation to the central lab. The string test was applied to obtain gastric juice for isolation of *H. pylori* as previously described,[34]. In brief: once extracted from the volunteers, the string was immediately inoculated on blood agar plates with antimicrobials, packed in Jars with Campy-pack CO_2_ generators (Beckton Dickinson Co., Sparks MD USA) and transported to the central lab for isolation of *H. pylori*.

### Genotyping of native individuals in blood samples

ABO and Rh(D) blood groups were determined in blood samples using commercial antiserum (Ortho Diagnostics, Raritan NJ). DNA was isolated from white blood cells and stored at −20°C until tested for mtDNA types and in the case of male volunteers for polymorphisms in the Y chromosome. Four different mtDNA regions were amplified by PCR using primers and conditions previously described [35], and cut with restriction enzymes: *HaeIII* for haplotype A, *HincII* for haplotype C, and *AluI* for haplotype D, and the resulting restriction fragments were analyzed by electophoresis in 2% agarose gels. The haplogroup B was analyzed in 8% polyacrylamide gels electrophoresis.

In order to study polymorphisms in Y-chromosome, two loci, *DYS19 and DYS199* were analyzed for a polymorphic tetranucleotide microsatellite, and for a biallelic marker (defined as M3) using primers and conditions reported previously [36]. The M3 PCR product was digested using *Mun*I, and **t**he resulting restriction fragments were analyzed by electrophoresis on polyacrylamide gels. In addition, DNA samples were studied for STRs markers, and D8S1179, D21S11, D7S820, CSF1PO, D3S1358, TH01, D13S317, D16S539, D2S1338, D19S433, vWA, TPOX, D18S51, D5S818 and FGA loci along with amelogenin gene fragment were co-amplified in a multiplex PCR reaction using the AmpFlSTR Identifiler TM Kit (Applied Biosystems, Foster City, CA) according to the manufacturer's protocols. The amplified products, together with reference allelic ladders were analyzed in an ABIPrism 3130 Genetic Analyzer (Applied Biosystems). Capillar electrophoresis results, as well as allele determination, were analyzed using GeneMapper1 software Version 3.5.

### *H. pylori* isolation and genotyping

The blood-agar plates with the string-extracted gastric juice were incubated at 37°C in a 10% CO_2_ atmosphere. From the primary growth, a single colony was picked up and propagated; and the 24 h growth was swept for DNA isolation. Primers used for amplification and sequencing of *vacA*, *cagA* and *hspA* are presented in Table 6. For sequencing, the *vacA*, *hspA*, and *cagA* PCR products were purified (Rapid Gel extraction systems, Marligen Bioscience, U.S.A.) and sequencing was performed by the dideoxynucleotide chain termination method with a BigDye Terminator Cycle Sequencing kit (Version 3.1, Applied Biosystems, Rockland, USA) in an ABI PRISM 377 automated DNA sequencer (Appplied Biosystems) as previously described [37]. The nucleotide sequences were analyzed by Chromas software (version 1.62; Technelysium) and aligned with DNAMAN program (Version 3.0, Lynnon BioSoft). All sequences were registered in GenBank and accession numbers are detailed in Table S2. Phylogenetic trees were constructed using the Neighbour-joining method with Kimura two-parameter distances, using MEGA V2.0 and 500 bootstrap samplings. PCR of representative *H. pylori* housekeeping genes, sequencing of PCR products and phylogenetic analyses of sequences obtained (MLST; multilocus sequence typing) were carried out as described [32]. The housekeeping gene sequences from strains of countries other than Mexico, included here for comparison, are the same as those used in ref 32.

**Table 6:** Primers used for amplification and sequencing of *vacA, cagA* and *hspA* genes.

TargetSequence	Primerdesignation	Use	Sequence
*vacA s*	VA1-F	Amplification	5′ATGGAAATACAACAAACACAC3′
	VA1-R	and sequencing	5′CTGCTTGAATGCGCCAAAC3′
*vacA m*	VAGF VAGR	Amplification	5′CAATCTGTCCAATCAAGCGAG3′
			5′GCGTCAAAATAATTCCAAGG3′
*5′cagA*	F1	Amplification	5′GATAACAGGCAAGCTTTTTGAGG3′
	B1		5′CTGCAAAAGAATGTTTGGCAG3′
*3′cagA*	CAGTF	Amplification	5′ACCCTAGTCGGTAATGGGTTA3
	CAGTR	and sequencing	´5′GTAATTGTCTAGTTTCGC3′
*hspA*	hspAF	Amplification and	5′TGCGCTATAGTTGTGTCGC3′
	hspAR	sequencing	5′GCTATCTGAAAATTTGATTTCTTTTGC3′

### Microarray Experiments

The microarray used represents the superset of genes present in *H. pylori* strains 26695 and J99 whole genome sequences, as previously described [38]. For the test, 0.5 µg of each of 26695 and J99 genomic DNA were denatured and five µl of 10X Buffer (400 µg/ml random octamers, 0.5 M Tris-HCL, 100 mM MgSO4 and 10 mM DTT), 5 µl dNTP/dUTP mix (0.5 mM dGTP, dATP, dCTP, 0.2 mM aminoallyl dUTP and 0.3 mM dTTP) and 1μl Klenow were added and the reaction incubated overnight. Free amines were removed and the probe (mixed J99 and 26695) was labelled with Cy3 dye (Amersham). One µg of genomic DNA from each test strain was labeled with Cy5 dye (Amersham, Pharmacia). Labeled probe and test DNA were combined and unincorporated dye removed, and 1 µl of 10 mg/ml yeast tRNA, 1.5 µl of 20X SSC and 1.5 µl of 1% SDS were added. The mixture was denatured, applied to the microarray slide and incubated overnight. The microarray slide was washed with 2X SSC and 0.1% SDS and then with 1X SSC for 5 minutes, three times. The microarray was scanned using an Axon scanner with GENEPIX 3.0 software (Axon Instruments, Redwood City, CA) and data were normalized and processed as previously described [38]. The mean of the normalized red/green (R/G) ratio was calculated using data from two arrays per isolate, yielding four readings for each gene. The cut off for absence of a gene was defined as a log2 (red/green) of <−1.0 based on test hybridizations using the sequenced strain J99 against the 26695/J99 mixed reference. The false positive and false negative rates were determined to be 3.5% and 0.34%, respectively [39]. All data is MIAME compliant and the raw data has been deposited in GEO (number GSM609344).

### Activity on AGS cells

For the cell assay 1×10^5^ AGS cells/ml were grown in 6-wells plates with F12 medium and 10% fetal bovine serum during 48 h. *H. pylori* strains to be tested were grown for 48 h in blood agar plates and a single colony was re-seeded on agar plate and incubated for growth during 24 h. *H. pylori* growth was harvested, and suspended in serum free F-12 medium, to reach an optical density of 0.1 at 550 nm (1.2×10^8^ bacteria/ml) before addition to AGS cells at a multiplicity of infection (MOI) of 1∶100 (cell:bacteria). *H. pylori* strain 26695 was used as positive control [20]. To monitor morphologic effects, co-cultures in 6-well plates was incubated for 24 hs, and observed for morphological changes in a microscope. The pattern of adhesion of *H. pylori* to cells was observed after 3 hs of co-culture, and induction of IL-8 was determined after incubating co-cultures for 6 hs, after this time concentration of IL-8 in the culture media was estimated using a commercial ELISA assay (BD Biosciences, San Diego, CA).

## Supporting Information

Figure S1 A map of Mexico with the location of the three Native groups studied, and location of the closest city for reference.
(TIFF)

Table S1 Describes the STR alleles present in thd individuals from the three Native Mexican groups studied, Nahua, Tarahumara and Huichol.
(DOC)

Table S2 Describes the GenBank accession numbers of the sequences from Mexican Native strains reported in this study.
(XLSX)

Table S3 Concordance analyses in the content of variable genes in the plasticity zones between the 368H Huichol and mestizo strains from patients with gastric cancer, gastritis and duodenal ulcer.
(DOC)
