# Supplementary figures and images for: Helicobacter pylori Genotyping from American Indigenous Groups Shows Novel Amerindian vacA and cagA Alleles and Asian, African and European Admixture
Source: PLoS One. 2011 Nov 3;6(11):e27212. doi: 10.1371/journal.pone.0027212 (PMC3207844; doi:10.1371/journal.pone.0027212)

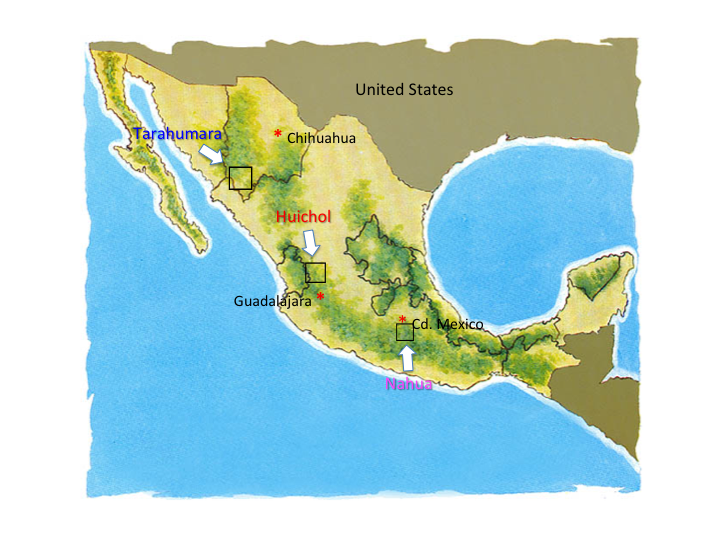

Supplement: Figure S1 — A map of Mexico with the location of the three Native groups studied, and location of the closest city for reference. (TIFF) [file pone.0027212.s001.tiff]
