# Supplementary material for: Helicobacter pylori Genotyping from American Indigenous Groups Shows Novel Amerindian vacA and cagA Alleles and Asian, African and European Admixture
Source: PLoS One. 2011 Nov 3;6(11):e27212. doi: 10.1371/journal.pone.0027212 (PMC3207844; doi:10.1371/journal.pone.0027212)
Supplement: Table S1 — Describes the STR alleles present in thd individuals from the three Native Mexican groups studied, Nahua, Tarahumara and Huichol. (DOC) [file pone.0027212.s002.doc]

Suplemental Table 1. STR alleles present in each of the individuals with *H. pylori* isolates from Nahua, Tarahumara and Huchol Native Mexican groups.

| Case | D8S1179 | D21S11 | D7S820 | CSF1PO | D3S1358 | TH01 | D13S317 | D16S539 | D2S1338 | D19s433 | vWA | TPOX | D18S51 | AMEL | D5S818 | FGA |
| --- | --- | --- | --- | --- | --- | --- | --- | --- | --- | --- | --- | --- | --- | --- | --- | --- |
| 2921N | 13 | 30 | 11 | 11 | 14 | 7 | 9 | 10 | 23 | 13.2 | 17 | 8 | 15 | X | 11 | 24 |
|  | 13 | 31 | 12 | 12 | 16 | 7 | 10 | 10 | 23 | 15 | 20 | 11 | 15 | Y | 11 | 26 |
| 172N | 12 | 30 | 11 | 11 | 15 | 5.3 | 9 | 11 | 17 | 13 | 16 | 8 | 17 | X | 9 | 22 |
|  | 13 | 31 | 11 | 12 | 17 | 6 | 10 | 12 | 18 | 14.2 | 17 | 8 | 17 | X | 11 | 24 |
| 50N | 10 | 29 | 10 | 12 | 15 | 6 | 9 | 11 | 22 | 13 | 17 | 8 | 16 | X | 11 | 22 |
|  | 15 | 30 | 13 | 12 | 16 | 7 | 11 | 13 | 22 | 14 | 17 | 8 | 17 | Y | 12 | 24 |
| 23O | 10 | 29 | 8 | 12 | 15 | 6 | 11 | 10 | 20 | 13 | 17 | 8 | 17 | X | 11 | 21 |
|  | 13 | 31.2 | 10 | 12 | 16 | 6 | 13 | 12 | 23 | 14 | 17 | 8 | 17 | X | 13 | 26 |
| 111N | 11 | 30.2 | 11 | 10 | 15 | 6 | 9 | 12 | 17 | 14 | 16 | 8 | 11 | X | 11 | 24 |
|  | 13 | 31.2 | 12 | 12 | 15 | 9.3 | 9 | 12 | 23 | 15 | 18 | 12 | 14 | Y | 11 | 25 |
| 11N | 13 | 29 | 12 | 12 | 15 | 7 | 9 | 11 | 19 | 13.2 | 15 | 8 | 14 | X | 11 | 24 |
|  | 15 | 31.2 | 12 | 12 | 16 | 9.3 | 9 | 11 | 24 | 15.2 | 15 | 11 | 15 | X | 12 | 26 |
| 193N | 12 | 29 | 11 | 11 | 15 | 7 | 9 | 12 | 18 | 14.2 | 16 | 8 | 13 | X | 11 | 19 |
|  | 14 | 31 | 11 | 12 | 16 | 7 | 10 | 12 | 19 | 15 | 18 | 8 | 17 | Y | 13 | 24 |
| 203N | 14 | 31.2 | 11 | 10 | 15 | 6 | 11 | 10 | 17 | 15.2 | 17 | 8 | 14 | X | 11 | 23 |
|  | 15 | 31.2 | 12 | 11 | 16 | 7 | 13 | 13 | 22 | 16.2 | 19 | 11 | 17 | Y | 12 | 25 |
| 2132N | 13 | 28 | 12 | 10 | 15 | 6 | 10 | 12 | 20 | 13 | 16 | 8 | 14 | X | 7 | 20 |
|  | 14 | 33.2 | 12 | 12 | 15 | 7 | 11 | 13 | 24 | 15 | 17 | 11 | 17 | Y | 11 | 25 |
| 58N | 13 | 30 | 10 | 12 | 15 | 6 | 12 | 10 | 19 | 13 | 16 | 11 | 13 | X | 11 | 19 |
|  | 13 | 30 | 11 | 12 | 16 | 7 | 13 | 13 | 23 | 13.2 | 19 | 12 | 17 | Y | 11 | 21 |
| 1831N | 10 | 29 | 11 | 12 | 15 | 6 | 8 | 10 | 18 | 13 | 16 | 8 | 12 | X | 7 | 19 |
|  | 15 | 29 | 11 | 14 | 15 | 7 | 11 | 13 | 19 | 15.2 | 16 | 11 | 16 | X | 12 | 19 |
| 203N | 13 | 30 | 10 | 10 | 15 | 5.3 | 8 | 12 | 18 | 13 | 15 | 8 | 13 | X | 10 | 21 |
|  | 14 | 33.2 | 11 | 11 | 17 | 8.3 | 12 | 12 | 23 | 15.2 | 16 | 8 | 14 | X | 12 | 24 |
| 2161N | 13 | 31.2 | 10 | 11 | 15 | 6 | 9 | 12 | 19 | 13.2 | 16 | 11 | 14 | X | 11 | 19 |
|  | 14 | 32.2 | 11 | 14 | 16 | 7 | 12 | 13 | 23 | 15 | 20 | 11 | 17 | Y | 11 | 19 |
| 10N | 13 | 30 |  |  |  |  |  |  |  |  |  |  |  |  |  |  |
|  | 17 | 30 |  |  |  |  |  |  |  |  |  |  |  |  |  |  |
| 632N | 10 | 29 | 11 | 11 | 15 | 5.3 | 9 | 11 | 23 | 13.2 | 18 | 8 | 12 | X | 11 | 19 |
|  | 14 | 34.2 | 11 | 12 | 15 | 7.3 | 12 | 12 | 24 | 14.2 | 18 | 11 | 14 | Y | 12 | 24 |
| 35N | 14 | 29 |  |  | 16 | 6.3 | 11 | 9 | 24 | 13 | 16 | 8 |  | X | 11 |  |
|  | 15 | 32.2 |  |  | 17 | 7 | 13 | 12 | 25 | 16.2 | 17 | 11 |  | X | 13 |  |
| 2133N | 13 | 30 | 10 | 10 | 15 | 6.3 | 9 | 10 | 23 | 14 | 16 | 11 | 13 | X | 11 | 19 |
|  | 13 | 31 | 11 | 12 | 16 | 7 | 13 | 13 | 24 | 15 | 19 | 12 | 17 | X | 11 | 21 |
| 373H | 13 | 30 | 11 |  | 15 | 6 | 9 |  | 22 | 14 | 16 | 12 | 17 | X | 10 | 22 |
|  | 14 | 30 | 11 |  | 15 | 7 | 11 |  | 23 | 15 | 16 | 12 | 18 | X | 11 | 25 |
| 368H | 13 | 31 | 12 | 10 | 15 | 6 | 9 | 10 | 19 | 13.2 | 16 | 8 | 14 | X | 12 | 19 |
|  | 13 | 31.2 | 13 | 11 | 17 | 8 | 9 | 11 | 20 | 14 | 19 | 12 | 19 | Y | 12 | 23 |
| 370H | 13 | 31.2 | 10 | 10 | 16 | 6 | 9 | 12 | 22 | 14 | 16 | 8 | 18 | X | 11 | 19 |
|  | 15 | 32.2 | 12 | 12 | 16 | 7 | 10 | 12 | 23 | 14 | 19 | 11 | 18 | X | 11 | 22 |
| 369H | 13 | 29 | 11 | 10 | 15 | 6 | 9 | 10 | 20 | 13.2 | 17 | 8 | 14 | X | 7 | 21 |
|  | 14 | 30 | 11 | 12 | 15 | 7 | 14 | 10 | 22 | 14 | 18 | 12 | 15 | Y | 11 | 26 |
| 580T | 10 | 27 | 10 | 11 | 15 | 6.3 | 10 | 9 | 20 | 17.2 | 18 | 8 | 14 | X | 7 | 23 |
|  | 13 | 31.2 | 11 | 12 | 16 | 7 | 11 | 10 | 23 | 17.2 | 18 | 8 | 15 | Y | 11 | 24 |
| 584T | 10 | 30 | 11 | 12 | 15 | 6.3 | 9 | 9 | 20 | 13.2 | 14 | 8 | 13 | X | 7 | 19 |
|  | 14 | 33.2 | 12 | 13 | 15 | 7 | 14 | 12 | 21 | 17.2 | 17 | 8 | 15 | Y | 11 | 22 |
| 590T | 12 | 29 | 12 | 12 | 15 | 6.3 | 10 | 9 | 18 | 13 | N | 11 | 14 | X | 7 | 20 |
|  | 14 | 32.2 | 13 | 12 | 16 | 7 | 11 | 9 | 20 | 13.2 | N | 11 | 18 | Y | 11 | 26 |
| 594T | 10 | 30 | 12 | 10 | 15 | 6.3 | 12 | 11 | 19 | 13.2 | 15 | 8 | 13 | X | 7 | 19 |
|  | 13 | 32.2 | 12 | 12 | 15 | 7 | 14 | 12 | 22 | 16.2 | 18 | 11 | 16 | X | 11 | 26 |
| 595T | 10 | 30 | 11 | 12 | 15 | 6 | 10 | 9 | 22 | 15.2 | 16 | 8 | 13 | X | 11 | 22 |
|  | 15 | 32.2 | 12 | 13 | 17 | 7 | 11 | 12 | 22 | 16.2 | 16 | 12 | 13 | X | 12 | 27 |
| 597T | 13 | 29 | 11 | 12 | 16 | 6 | 10 | 12 | 18 | 15 | 15 | 8 | 13 | X | 11 | 19 |
|  | 14 | 30 | 13 | 12 | 17 | 7 | 13 | 12 | 19 | 16.2 | 18 | 12 | 15 | X | 13 | 28 |
| 599T | 13 | 29 | 11 | 10 | 15 | 6 | 10 | 11 | 19 | 13 | 14 | 8 | 13 | X | 11 | 26 |
|  | 14 | 29 | 12 | 12 | 16 | 7 | 10 | 12 | 20 | 16.2 | 16 | 11 | 16 | Y | 13 | 26 |
| 600T | 13 | 31.2 | 10 | 12 | 15 | 6 | 10 | 10 | 20 | 13.2 | 16 | 8 | 13 | X | 11 | 19 |
|  | 14 | 32.2 | 12 | 12 | 16 | 7 | 10 | 10 | 22 | 15.2 | 18 | 11 | 14 | X | 11 | 24 |
| 603T | 10 | 31.2 | 11 | 10 | 16 | 6.3 | 9 | 9 | 20 | 15 | N | 8 | 15 | X | 7 | 24 |
|  | 14 | 33.2 | 12 | 12 | 16 | 7 | 13 | 12 | 23 | 16.2 | 19 | 8 | 15 | X | N | 25 |
| 639T | 11 | 30 | 12 | 12 | 15 | 6.3 | 11 | 12 | 19 | 13.2 | 15 | 8 | 13 | X | 11 | 22 |
|  | 14 | 32.2 | 12 | 12 | 16 | 7 | 11 | 13 | 20 | 17.2 | 18 | 8 | 17 | X | 11 | 27 |
