# Supplementary material for: Helicobacter pylori Genotyping from American Indigenous Groups Shows Novel Amerindian vacA and cagA Alleles and Asian, African and European Admixture
Source: PLoS One. 2011 Nov 3;6(11):e27212. doi: 10.1371/journal.pone.0027212 (PMC3207844; doi:10.1371/journal.pone.0027212)
Supplement: Table S3 — Concordance analyses in the content of variable genes in the plasticity zones between the 368H Huichol and mestizo strains from patients with gastric cancer, gastritis and duodenal ulcer. (DOC) [file pone.0027212.s004.doc]

| Suplemental Table 3. Concordance analyses in the content of variable genes in the plasticity zones between the 368H Huichol and mestizo strains from patients with gastric cancer, gastritis and duodenal ulcer. | | | | | |
| --- | --- | --- | --- | --- | --- |
|  |  | **PZ1** | | **PZ2** | |
| **Disease** | **Mestizo isolates** | **Kappa*** | **p** | **Kappa*** | **p** |
| **Gasric cancer** | **c188S32** | --- | --- | 0.1102 | 0.0896 |
|  | **c206S32** | 0.2791 | 0.0597 | -0.0286 | 0.4117 |
|  | **c331S31** | 0.6421 | <.0001 | 0.1130 | 0.1635 |
|  | **c377S42** | -0.0758 | 0.2913 | -0.1475 | 0.0918 |
|  | **c401S41** | 0.1667 | 0.0352 | -0.0623 | 0.3071 |
|  | **c443S61** | 0.3115 | 0.0318 | 0.2895 | 0.0156 |
|  | **c456S62** | 0.5556 | 0.000432 | -0.0794 | 0.2372 |
|  | **c510S33** | 0.0427 | 0.2131 | 0.2523 | 0.0298 |
|  | **c510S34** | 0.0782 | 0.2504 | 0.0365 | 0.3930 |
|  | **c510S43** | 0.0556 | 0.1552 | 0.0424 | 0.3357 |
|  | **c510S44** | 0.6000 | <.0001 | 0.0025 | 0.4924 |
|  | **c535S41** | 0.5873 | <.0001 | 0.1935 | 0.0366 |
| **Gastritis** | **c103S31** | 0.4773 | 0.0110 | 0.4708 | 0.0006 |
|  | **c103S41** | --- | --- | -0.0334 | 0.2351 |
|  | **c110S33** | 0.0398 | 0.3653 | 0.0564 | 0.2969 |
|  | **c176S32** | 0.2397 | 0.1208 | -0.2027 | 0.0821 |
|  | **c221S31** | -0.4379 | 0.0014 | -0.0999 | 0.2512 |
|  | **c284S31** | 0.5746 | 0.0005 | 0.0955 | 0.2372 |
|  | **c284S41** | 0.4882 | 0.0011 | -0.1206 | 0.1898 |
|  | **c320S32** | 0.1250 | 0.2535 | -0.2160 | 0.0911 |
|  | **c320S41** | 0.4101 | 0.0030 | -0.2278 | 0.0249 |
|  | **c345S32** | 0.4242 | 0.0040 | 0.0899 | 0.2438 |
|  | **c357S31** | 0.5072 | 0.0002 | -0.0340 | 0.3710 |
|  | **c357S32** | 0.6961 | <.0001 | -0.1583 | 0.1081 |
|  | **c370S35** | 0.4022 | 0.0043 | -0.0470 | 0.0944 |
|  | **c386S33** | 0.4550 | 0.0044 | 0.4478 | <.0001 |
|  | **c386S36** | -0.1495 | 0.0681 | -0.3785 | 0.0012 |
|  | **c386S43** | --- | --- | 0.0301 | 0.3192 |
| **Duodenal ulcer** | **c174S32** | 0.3750 | 0.0314 | 0.3970 | 0.0244 |
|  | **c174S43** | 0.3862 | 0.0037 | 0.0714 | 0.2868 |
|  | **c204S43** | 0.4435 | 0.0110 | 0.1964 | 0.0558 |
|  | **c204S46** | 0.4502 | 0.0072 | 0.1619 | 0.0857 |
|  | **c261S32** | 0.6413 | <.0001 | -0.0203 | 0.4417 |
|  | **c263S41** | --- | --- | --- | --- |
|  | **c336S32** | 0.7021 | <.0001 | 0.3783 | 0.0049 |
|  | **c355S34** | 0.2708 | 0.1011 | -0.0368 | 0.2534 |
|  | **c362S36** | 0.6833 | <.0001 | 0.0178 | 0.4436 |
|  | **c372S32** | 0.4099 | 0.0134 | 0.0926 | 0.2672 |
|  | **c372S33** | 0.5119 | 0.0005 | 0.2514 | 0.0249 |
|  | **c379S42** | --- | --- | --- | --- |
|  | **c387S31** | -0.3570 | 0.0084 | -0.0698 | 0.0987 |
|  | **c387S42** | -0.4000 | 0.0032 | 0.1421 | 0.1174 |
| Kappa coefficient for concordance betwen presence- absence of the genes between the Huichol and mestizo isolates. A value of 1 indicate concordance, a value < 0 no concordance | | | | | |
